# A Comprehensive Study of Soft Palate Development in Mice

**DOI:** 10.1371/journal.pone.0145018

**Published:** 2015-12-15

**Authors:** Alexandre Grimaldi, Carolina Parada, Yang Chai

**Affiliations:** Center for Craniofacial Molecular Biology, Herman Ostrow School of Dentistry, University of Southern California, Los Angeles, California, United States of America; Laboratoire de Biologie du Développement de Villefranche-sur-Mer, FRANCE

## Abstract

Cleft palate is one of the most common congenital birth defects. Tremendous efforts have been made over the last decades towards understanding hard palate development. However, little is known about soft palate morphogenesis and myogenesis. Finding an appropriate surgical repair to restore physiological functions of the soft palate in patients with cleft palate is a major challenge for surgeons, and complete restoration is not always achievable. Here, we first analyzed the morphology, orientation and attachments of the four muscles of the murine soft palate and found that they are very similar to their counterparts in humans, validating the use of *mus musculus* as a model for future studies. Our data suggests that muscle differentiation extends from the lateral region to the midline following palatal fusion. We also detected an epithelial seam in the fusing soft palatal shelves, consistent with the process of fusion of the posterior palatal shelves, followed by degradation of the epithelial remnants. Innervation and vascularization are present mainly in the oral side of the soft palate, complementing the differentiated muscles. Cell lineage tracing using *Wnt1-Cre;Zsgreen* ^*fl/fl*^ mice indicated that all the tendons and mesenchyme embedding the soft palate muscles are neural crest-derived. We propose that the posterior attachment of the soft palate to the pharyngeal wall is an interface between the neural crest- and mesoderm-derived mesenchyme in the craniofacial region, and thus can serve as a potential model for the study of boundaries during development. Taken together, our study provides a comprehensive view of the development and morphology of the murine soft palate and serves as a reference for further molecular analyses.

## Introduction

The human secondary palate is divided into an anterior hard palate, consisting of the palatine processes of the maxillary bone and the palatine bone, and a posterior muscular soft palate. The hard palate provides a physical separation between the oral and nasal cavities. The soft palate muscles elevate or depress the palate and synergize with the pharyngeal muscles to close and open the oral and nasal cavities. These movements play important roles in speech, swallowing, breathing, and hearing.

Palatogenesis is a highly regulated sequence of developmental events that takes place between the 6^th^ and 12^th^ weeks of embryonic development in humans. During this process, the two palatal shelves elongate, elevate to acquire a horizontal conformation, and extend towards the midline where they meet and fuse. The medial edge epithelium degrades, leading to mesenchymal continuity [1, 2]. Any disturbance of these events can lead to a cleft palate, one of the most common birth defects in humans. Surgery can provide a partial solution by reconstructing the roof of the mouth, but often fails to completely restore the function of the soft palate for cases with larger defects [3, 4]. This usually translates into long-term impairment of quality of life due to abnormal soft palate muscle arrangement and velopharyngeal dysfunction. Different surgical techniques are utilized depending on the type of cleft and respiratory defect associated with it, in order to limit sleep apnea and velopharyngeal insufficiency and to improve phonation, all common issues for children with cleft palate [5–7].

In recent years, tremendous effort has been made to characterize the genetic regulators controlling the outgrowth, elevation and fusion of the palatal shelves. Although the development of the hard palate has been widely studied, little is known about the formation of the soft palate and the regulatory events controlling soft palate morphogenesis and myogenesis. Previous studies have suggested that the posterior palatal shelves elevate differently than the anterior hard palate [8, 9]. Some genes are expressed in specific regions along the antero-posterior axis in the developing palate, and their disruption can affect the anterior and posterior parts differentially [10–12]. Some studies have suggested that the soft palate forms through merging, as opposed to fusion, because an epithelial seam was not observed at the midline [13]. In contrast, other studies demonstrate the presence of an epithelial seam at the midline of the soft palate, supporting a model of fusion of the two palatal shelves, as in hard palate development. However, the degradation of this epithelial seam proceeds more rapidly than it does in the hard palate, making its visualization more difficult [14].

The anatomy of the human soft palate musculature has been well examined over the past decades. It is composed of five muscles: the tensor veli palatini (TVP), levator veli palatini (LVP), the palatoglossus (PLG), palatopharyngeus (PLP) and the musculus uvulae (absent in mice). Although mouse models have been widely used to study palatogenesis, few studies have compared mouse and human soft palates [14]. To date, little is known about mouse soft palate anatomy, with a few studies focusing on the TVP and LVP, but not on the PLG and PLP [15, 16]. We have previously described a mouse model for submucous cleft palate with cleft soft palate, *K14-Cre;Tgfbr2* ^*fl/fl*^ mice, with epithelium-specific deletion of *Tgfbr2*, a receptor for the TGFβ signaling pathway. The Wnt/β-catenin pathway is disrupted in the posterior palatal mesenchyme of these mice due to the upregulation of *Dkk1*, leading to a reduction in muscle mass, proliferation and differentiation [15]. Here, we focus on analyzing murine soft palate morphology in comparison to human anatomy, including the orientation and attachments of all four muscles of the mouse soft palate, as well as their appearance during palatal fusion. In addition, we highlight the development of soft palate muscles in relation to the surrounding nerves, vasculature, tendons, cranial neural crest (CNC)-derived mesenchyme and mesoderm-derived mesenchyme of the pharyngeal region. This study will serve as a strong foundation for further molecular analysis and mutant mouse characterizations, thus leading to better treatment and prevention of muscle defects associated with cleft palate.

## Materials and Methods

### Mouse lines

The studies were performed using C57BL/6 mice. In order to trace the CNC lineage, we crossed *Wnt1-Cre;Zsgreen* ^*fl/+*^ males with *Zsgreen* ^*fl/fl*^ females to generate *Wnt1-Cre;Zsgreen* ^*fl/fl*^ mice. Pregnant females were sacrificed at the indicated stage, as determined by counting the number of days after detection of a vaginal plug. The animals were euthanized through carbon dioxide overdose followed by cervical dislocation. All studies were performed with the approval of the Institutional Animal Care and Use Committee (IACUC) of the University of Southern California.

### Embryo processing

Embryos were fixed overnight in 4% paraformaldehyde and processed through a routine dehydration procedure for paraffin embedding. 10μm-thick paraffin sections were then either stained using hematoxylin-eosin or processed for immunofluorescence staining, after undergoing antigen retrieval. *Wnt1-Cre;Zsgreen* ^*fl/fl*^ samples were embedded in OCT after overnight immersion in 20% sucrose and prepared for frozen sectioning. The same samples underwent antigen retrieval and classic immunofluorescence staining following standard procedures. All analyses were performed in triplicate.

### Immunofluorescence staining

The following antibodies were used for this study: mouse monoclonal to MHC (Developmental Studies Hybridoma Bank: MF20), mouse monoclonal to β3-Tubulin (abcam: #ab78078), rabbit polyclonal to Col1a1 (abcam: #ab21286), and rabbit polyclonal to CD31 (abcam: #28364). Secondary antibodies used were polyclonal goat anti-mouse and anti-rabbit, coupled with Alexa Fluor^®^ 488 and Alexa Fluor^®^ 568 (Life Technologies: #A11001, #A11004, #A11008, A#11011). Staining was performed according to standard procedures. Due to the species of the antibodies used, the neuro-vasculature analysis was performed using adjacent sections aligned and merged using Adobe Photoshop CS4. The immunofluorescent sections were visualized using Leica DMI 3000B and Keyence BZ-X710 microscopes.

## Results

### Murine and human soft palates are highly homologous

**Fig 1:**
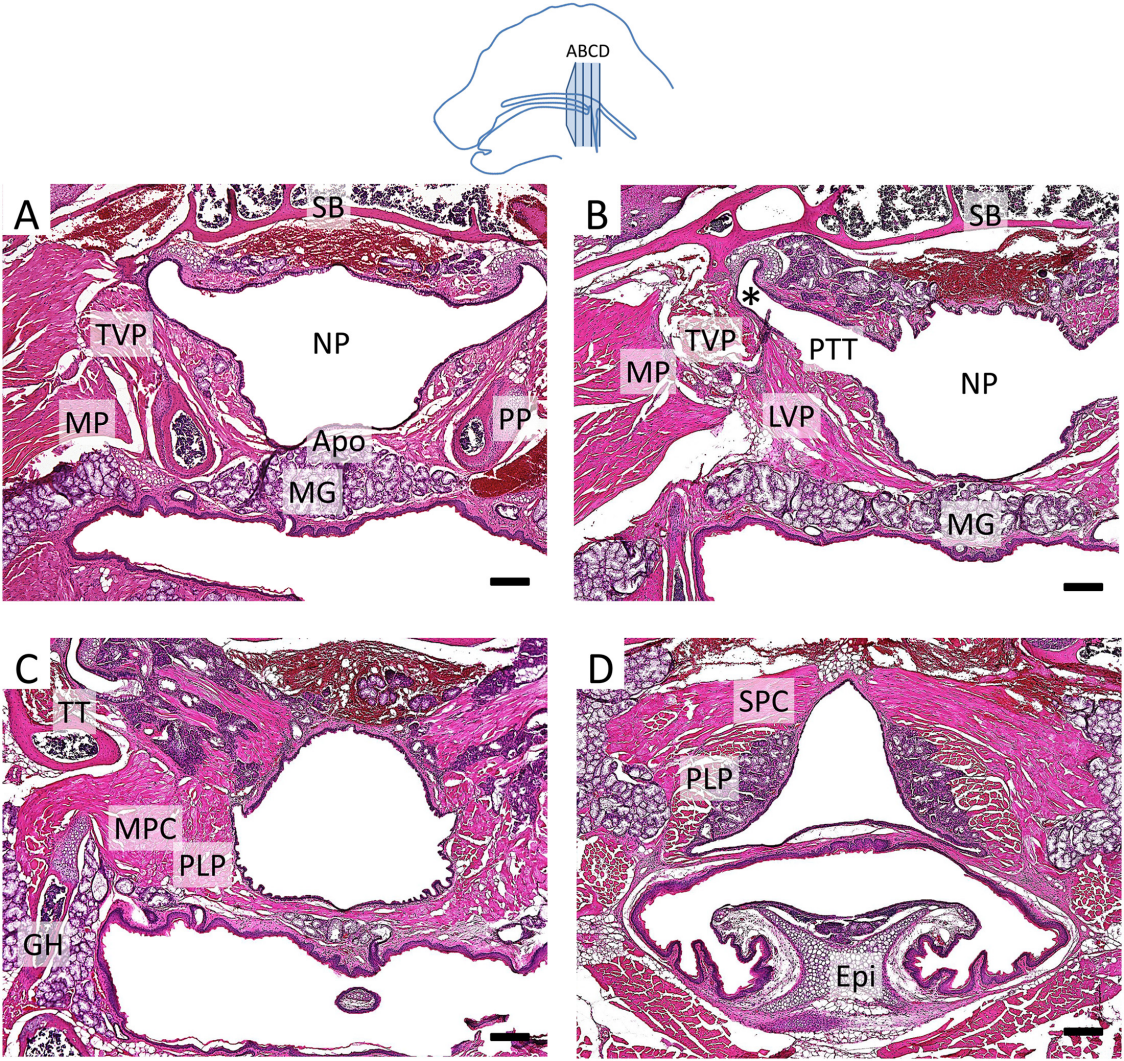
Murine and human adult soft palates are highly homologous. **(A-D)** Histological sections of a 4-month-old mouse soft palate stained with hematoxylin & eosin at the level of the TVP (A), LVP (B), MPC and PLP (C), and SPC and PLP (D). *: pharyngotympanic tube cartilage, Apo: palatine aponeurosis, Epi: epiglottis, GH: greater horn of the hyoid bone, LVP: levator veli palatini, MG: mucous glands, MP: medial pterygoid muscle, MPC: middle pharyngeal constrictor, NP: nasopharynx, PLP: palatopharyngeus, PP: pterygoid plate, PTT: pharyngotympanic tube, SB: sphenoid bone, TT: tensor tympani, TVP: tensor veli palatini. The schematic drawing indicates the orientation and the position of each section. Scale bars: 200μm.

**Fig 2:**
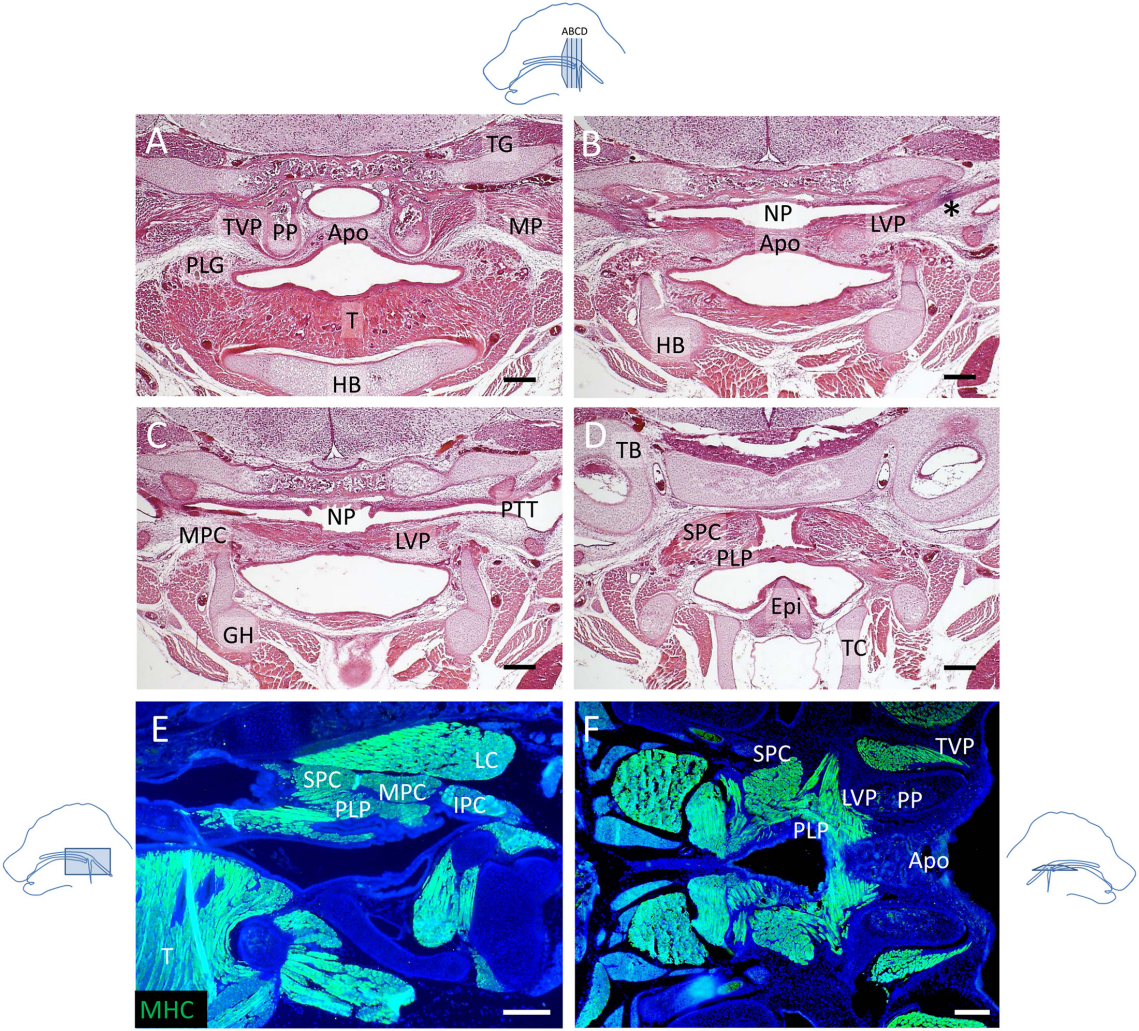
The morphology and attachment points of soft palate muscles in newborn mice appear fully developed. **(A-D)** Histological sections of soft palates from newborn mice stained with hematoxylin & eosin at the level of the TVP and PLG (A), TVP and LVP (B), LVP and MPC (C), and SPC and PLP (D). **(E, F)** MHC immunostaining (green) of sagittal (E) and transverse (F) sections of soft palates from newborn mice. *: tendon of the TVP, Apo: palatine aponeurosis, Epi: epiglottis, GH: cartilage primordium of the greater horn of the hyoid bone, HB: cartilage primordium of the hyoid bone, IPC: inferior pharyngeal constrictor, LC: longus capitis, LVP: levator veli palatini, MP: medial pterygoid muscle, MPC: middle pharyngeal constrictor, NP: nasopharynx, PLG: palatoglossus PLP: palatopharyngeus, PP: pterygoid plate, PTT: pharyngotympanic tube, SPC: superior pharyngeal constrictor, T: tongue, TC: thyroid cartilage, TG: trigeminal ganglion, TVP: tensor veli palatini. The schematic drawings indicate the orientation and the position of each section. Scale bars: 200μm.

In order to investigate soft palate anatomy in mice, we analyzed adult mice using histochemistry (Fig 1) and newborn mice using both histochemistry and myosin heavy chain (MHC) immunostaining (Fig 2). We found that the orientation of the soft palate muscles and the attachments of the soft palate and pharyngeal muscles in mice were homologous to that of humans [17]. The aponeurosis consisted of a thin layer of fibrous tissue overlaying an extensive group of mucous glands on the oral side of the adult soft palate (Fig 1A). At the newborn stage, the soft palate contained significantly fewer mucous glands and mainly consisted of undifferentiated mesenchyme (Fig 2A). The TVP was located on the outer side of the pterygoid plate (PP), and the LVP was located on the nasal side of the palate, above the PP. Moreover, the TVP originated from the scaphoid fossa of the pterygoid plate and attached to the sphenoid bone and cartilage of the pharyngotympanic tube, whereas the LVP attached to the cartilage of the pharyngotympanic tube and petrous part of the temporal bone (Figs 1B and 1C and 2B). The PLG attached to the side of the tongue (Fig 2A). Likewise, the middle pharyngeal constrictor (MPC) attached to the greater horns of the hyoid bone (Figs 1C and 2C) and its fibers were adjacent to those of the PLP, which attached posteriorly to the pharyngeal wall (Figs 1D and 2D–2F). In humans, the PLP and the superior pharyngeal constrictor (SPC) form a sheet that may act as a sphincter in the pharyngeal region [18]. Likely due to this intricate relationship, the PLP and SPC were difficult to distinguish in coronal view (Fig 2D) but could be visualized as more clearly related in transverse and sagittal views (Fig 2E and 2F). The soft palate muscles attached to the aponeurosis anteriorly (Fig 2E). These anatomical characteristics are all common to both humans and mice, which suggests that the morphological differences between mice and humans do not derive from a muscular rearrangement.

### Muscle differentiation extends from the lateral region to the midline following palatal fusion

**Fig 3:**
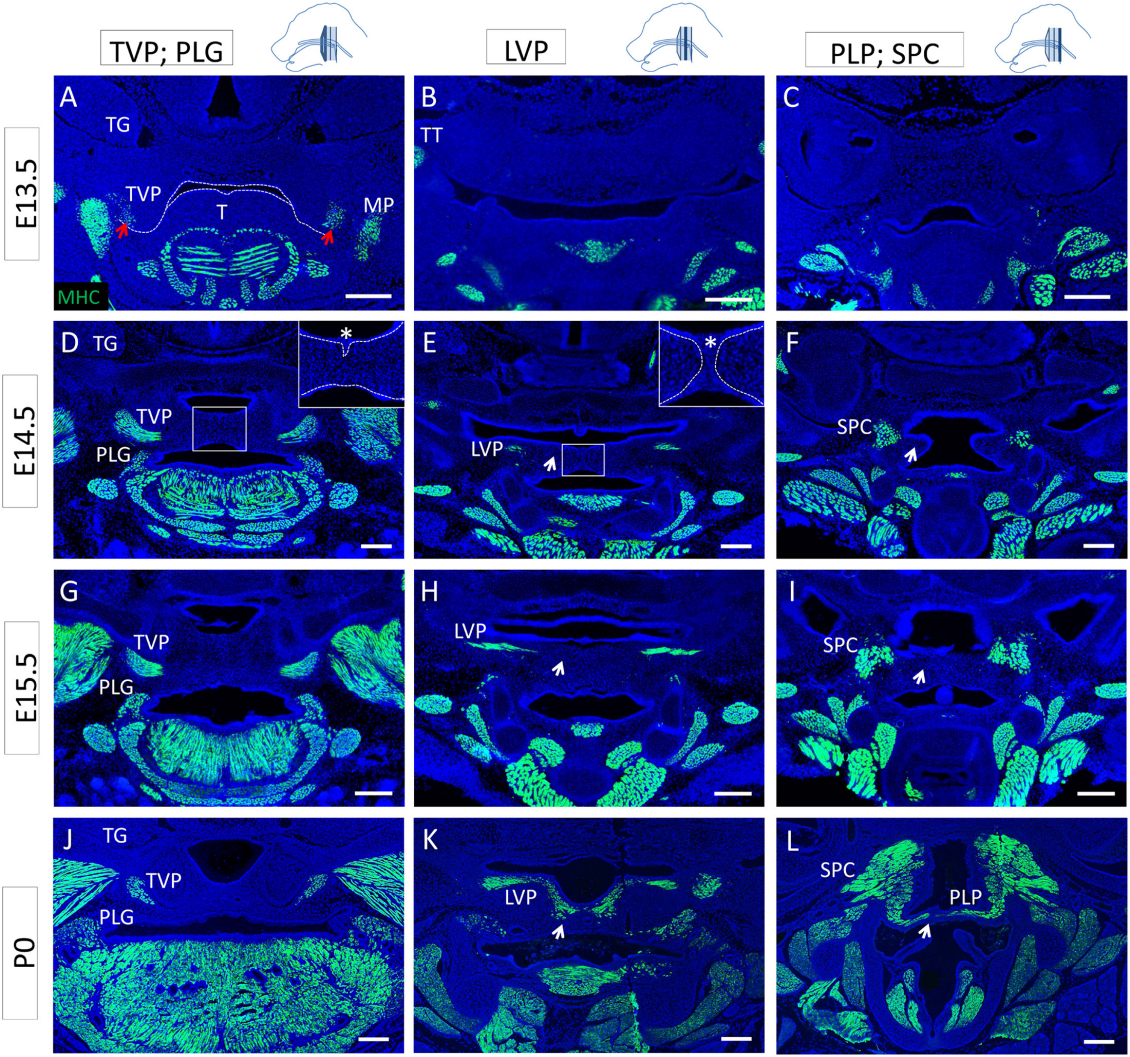
Differentiation of the soft palate muscles proceeds from the lateral regions to the midline. **(A-L)** MHC (green) along the antero-posterior axis of the mouse soft palate at E13.5 (A-C), E14.5 (D-F), E15.5 (G-I), and P0 (J-L) at the level of the TVP and PLG (A, D, G, J), LVP (B, E, H, K), and PLP and SPC (C, F, I, L). (A) Dashed lines indicate the outline of the palatal shelf and tongue epithelium. Red arrows indicate the differentiated TVP. (D-E) In the inset, dashed lines correspond to the basal lamina of the palatal shelf epithelium, and the asterisk indicates the epithelial seam during the fusion process. Arrows indicate the medial region of the palatal shelf negative for mature myocytes until newborn stage. LVP: levator veli palatini, MP: medial pterygoid muscle, PLG: palatoglossus PLP: palatopharyngeus, SPC: superior pharyngeal constrictor, T: tongue, TT: tensor tympani, TG: trigeminal ganglion, TVP: tensor veli palatini. The schematic drawings indicate the orientation and the position of each section. Scale bars: 200μm.

Recent studies have described the commitment of mesenchymal subpopulations during palatogenesis and the differentiation of the osteogenic lineage in the nasal side of the palatal shelves after fusion [12], but little is known about the differentiation pattern of the muscle compartment during these morphogenetic events. Therefore, we analyzed the differentiation status of the soft palate muscles before, during and after fusion and at newborn stage using MHC immunostaining. At E13.5, before palatal shelf fusion, the soft palatal shelves appeared anteriorly as two small protrusions flanking the tongue, whereas no clear structure was identifiable posteriorly (Fig 3A–3C). At this stage, the TVP expressed MHC (Fig 3A), but we failed to detect MHC expression in the other palatal muscles (Fig 3B and 3C). The tensor tympani (TT) was also clearly differentiated. At E14.5, as the soft palatal shelves were in the process of fusing, the anterior part of the soft palatal shelves had reached the midline and the epithelial seam was starting to disintegrate (Fig 3D). The TVP and PLG appeared differentiated and morphologically similar to their state at newborn stage. More posteriorly, the palatal shelves were in contact and a clear epithelial seam was detectable at the midline (Fig 3E). The LVP only expressed MHC in the lateral region. At the level of the PLP, the palatal shelves had not yet made contact, and, although the pharyngeal constrictor muscles were differentiated, no differentiated PLP was identifiable (Fig 3F). At E15.5, fusion was complete; we detected mesenchymal confluence all along the antero-posterior axis and no epithelial seam (Fig 3G–3I). MHC expression in the TVP and PLG at E15.5 was similar to E14.5, whereas it was extended in the lateral region of the LVP but not in the midline (Fig 3H). Because of the close relationship between the PLP, SPC and MPC, the lateral fibers of the PLP were not distinguishable from the pharyngeal constrictor muscles, making it difficult to assess its differentiation state. However, MHC staining was clearly not detectable in the medial region (Fig 3I). At newborn stage, all muscles exhibited their final morphology, and the LVP and PLP appeared continuously differentiated along the latero-medial axis. This spatio-temporal pattern of muscle differentiation suggests that undifferentiated myoblasts in the midline of the LVP and PLP fuse and differentiate as palatal shelf fusion is completed, leading to continuous muscle fibers at newborn stage. Moreover, *MyoD-Cre;Zsgreen* ^*fl/fl*^ cell lineage tracing indicated that the midline region was depleted of myoblasts at E15.5 (data not shown), suggesting that the newly differentiated muscle originated from the lateral regions. Thus, we hypothesize that an undifferentiated subpopulation of myogenic progenitors is present in the lateral region alongside the differentiated muscle and will progressively migrate and differentiate towards the midline of palate.

### Innervation and vascularization are present in the oral side of the palatal shelves

**Fig 4:**
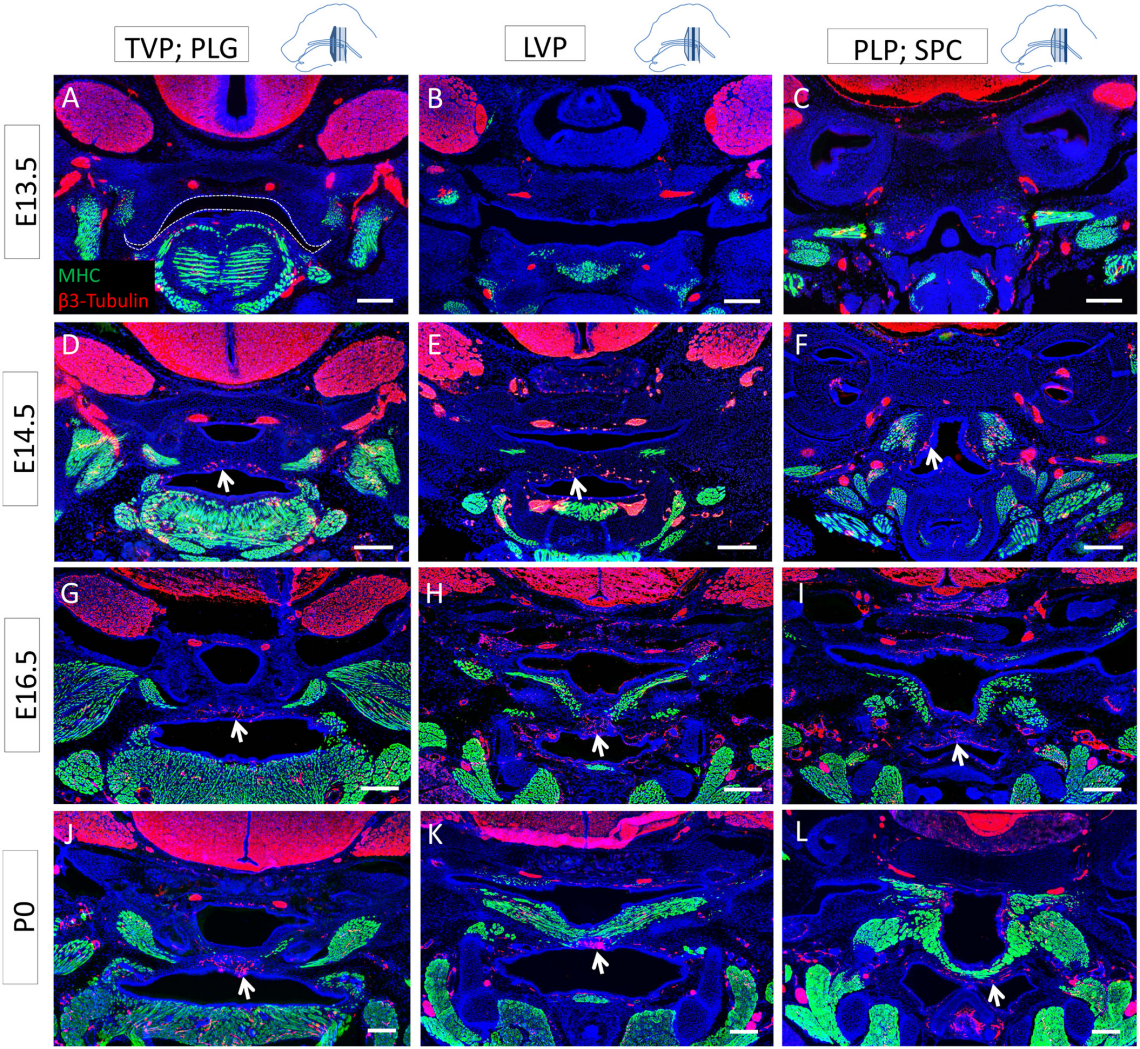
Soft palate innervation is primarily oro-medial and complements the differentiated muscle pattern. **(A-L)** MHC (green) and β3-tubulin (red) co-immunostaining along the antero-posterior axis of the mouse soft palate at E13.5 (A-C), E14.5 (D-F), E16.5 (G-I), and P0 (J-L) at the level of the TVP and PLG (A, D, G, J), LVP (B, E, H, K), and PLP and SPC (C, F, I, L). Dashed lines indicate the outline of the palatal shelf and tongue epithelium. Arrows indicate the nerve fibers innervating the palatal shelves in a pattern complementary to the differentiated muscle, mainly located orally in the medial region. The schematic drawings indicate the orientation and the position of each section. Scale bars: 200μm.

**Fig 5:**
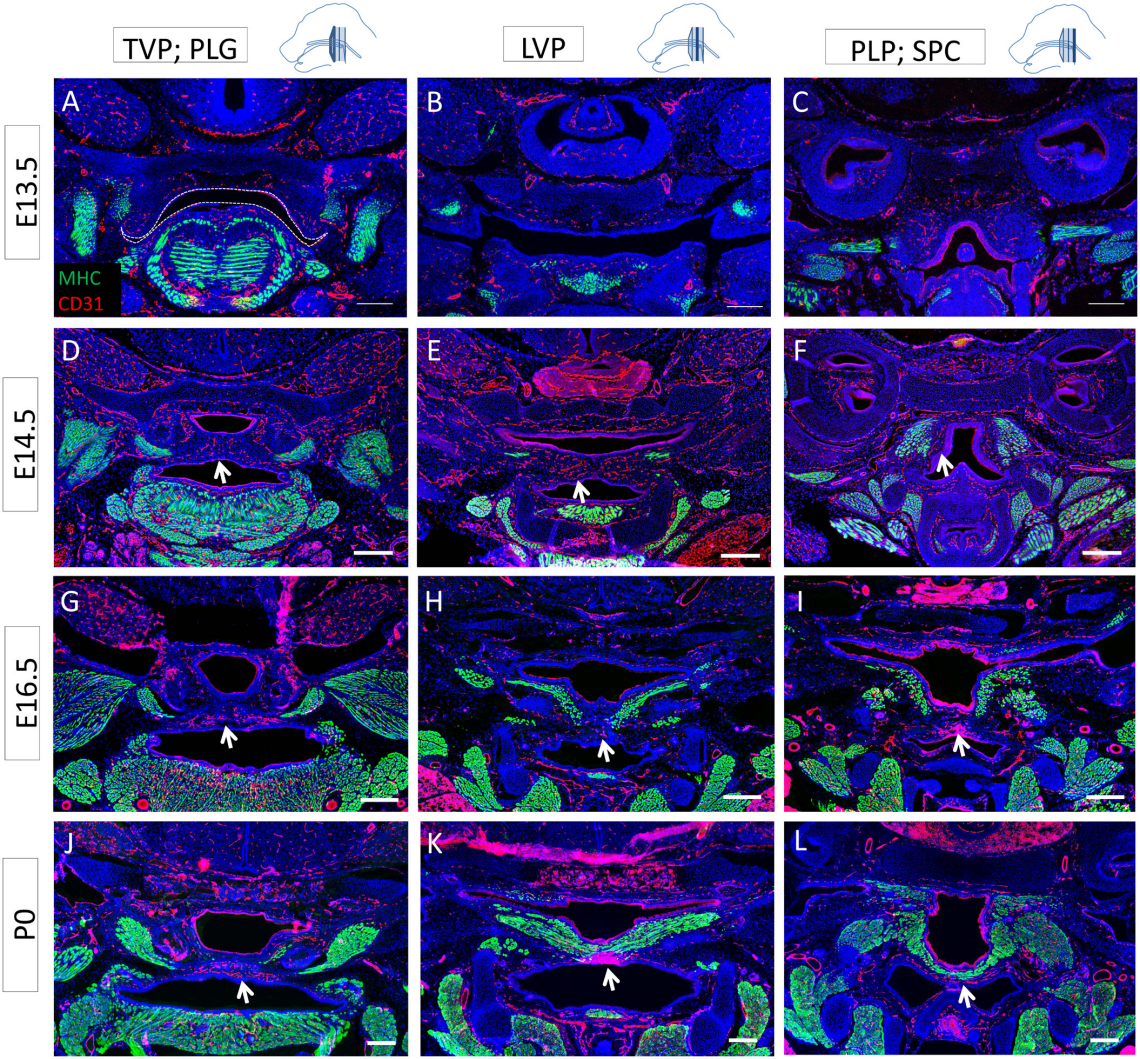
Soft palate vascularization is closely related to innervation and located mainly in oro-medial regions. **(A-L)** MHC (green) and CD31 (red) co-immunostaining along the antero-posterior axis of the mouse soft palate at E13.5 (A-C), E14.5 (D-F), E16.5 (G-I), and P0 (J-L) at the level of the TVP and PLG (A, D, G, J), LVP (B, E, H, K), and PLP and SPC (C, F, I, L). Dashed lines indicate the outline of the palatal shelf and tongue epithelium. Arrows indicate blood vessels supplying the palatal shelves in a pattern similar to that of innervation and complementary to the differentiated muscle, mainly located orally in the medial region. The schematic drawings indicate the orientation and the position of each section. Scale bars: 200μm.

Previous studies have shown that vasculature and innervation have important roles in regulating organogenesis and tissue homeostasis [19–21]. Therefore, we investigated the neuro-vascular patterns in regards to the differentiated muscles of the developing soft palate. At E13.5, we detected innervation in the differentiated TVP and tensor tympani but not the anterior palatal mesenchyme (Fig 4A and 4B). Both the palatal shelves and pharyngeal wall were vascularized (Fig 5A–5C). Posteriorly, the palatal shelves were not detectable, but the pharyngeal wall was also innervated (Fig 4C). The medial region of the palatal shelves was abundantly innervated and vascularized from E14.5 to newborn stage (Figs 4D–4L and 5D–5L). In E14.5 samples in which the palatal shelves had not yet made contact, extensive innervation was visible in the medial regions, underlying the epithelium (S1 Fig). At all stages, most of the nerves and blood vessels were located in the oral side of the palatal shelves, in close relationship to each other, underneath the muscle. From E16.5 on, the neuro-vasculature and the differentiated muscle patterns appeared complementary, with nerves and blood vessels mainly located in the medial region, in the oral side of the palatal shelves, lacking differentiated muscle (Figs 4G–4L and 5G–5L). This pattern could suggest a putative role for the nerves and vasculature in regulating morphogenesis of the soft palate, migration of myoblasts from the lateral regions to the midline, and/or differentiation.

### The pharyngeal region constitutes a boundary between neural crest cell-derived and mesoderm-derived mesenchyme in mice

**Fig 6:**
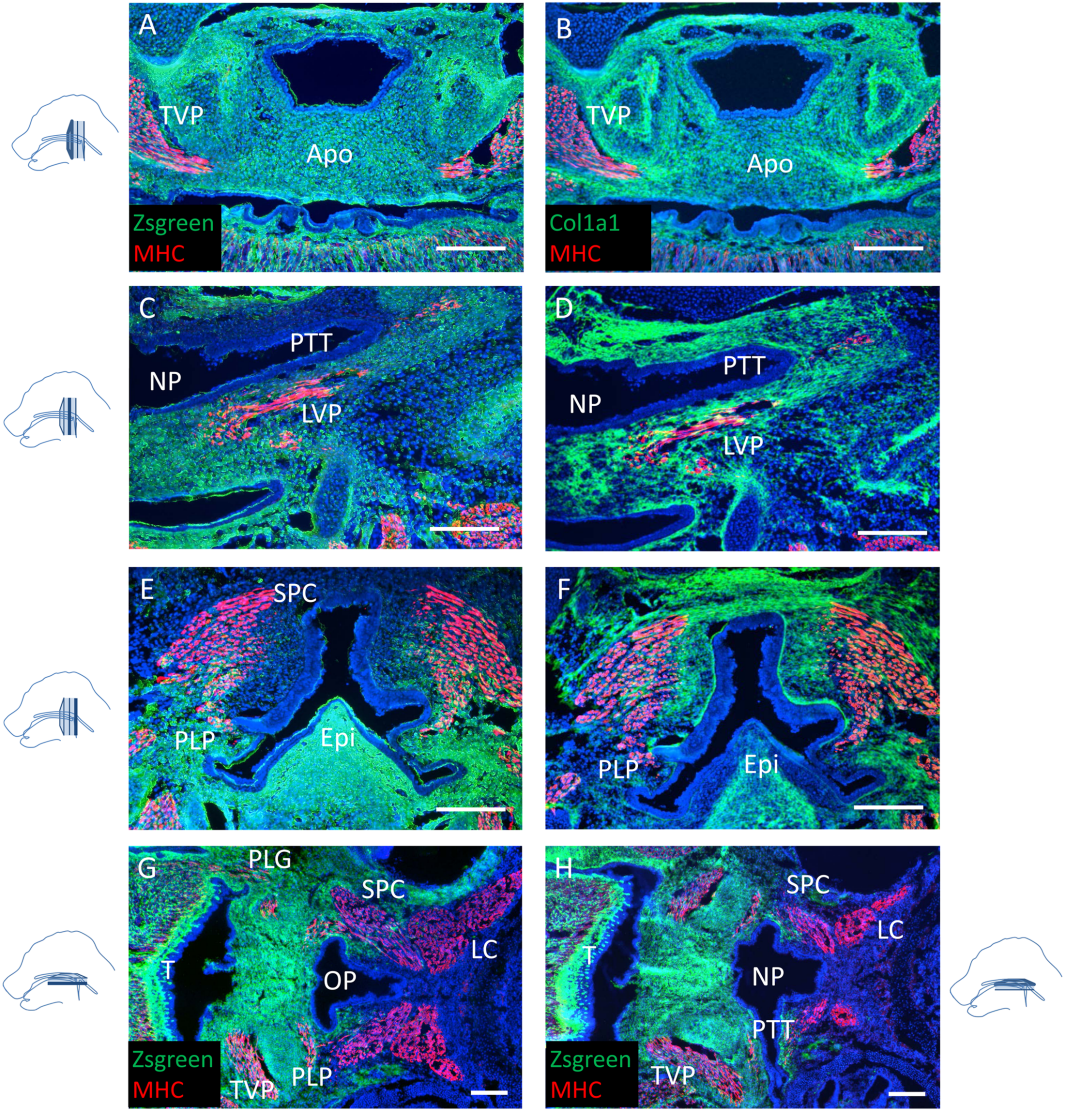
The pharyngeal region constitutes an anatomical boundary between neural crest- and mesoderm-derived tissues. **(A-F)** MHC (red) immunostaining and co-immunostaining with Col1a1 (green) of coronal sections of soft palates from E15.5 *Wnt1-Cre;Zsgreen* ^*fl/fl*^ mice. Positive ZsGreen signal indicates cells are neural crest-derived. **(G, H)** MHC (red) immunostaining of transverse sections of soft palates from E18.5 *Wnt1-Cre;Zsgreen* ^*fl/fl*^ samples mice at the level of the PLG (G) and the pharyngotympanic tube (H). Apo: palatine aponeurosis, Epi: epiglottis, LC: longus capitis, LVP: levator veli palatini, NP: nasopharynx, OP: oropharynx, PLG: palatoglossus PLP: palatopharyngeus, PTT: pharyngotympanic tube, SPC: superior pharyngeal constrictor, T: tongue, TVP: tensor veli palatini. The schematic drawings indicate the orientation and the position of each section. Scale bars: 200μm.

To identify the contribution of the CNC in the developing soft palate and pharynx, we performed cell lineage tracing utilizing E15.5 *Wnt1-Cre;Zsgreen* ^*fl/fl*^ mice immunostained for type I collagen (Col1a1) and MHC. Col1a1 is a marker for structural extracellular matrix, highlighting the orientation of the attachment of the soft palate muscles. As previously reported [16], the aponeurosis was CNC-derived (Fig 6A and 6B). Additionally, all the mesenchyme and tendons of the soft palate muscles were derived from the cranial neural crest. The oral, but not the nasal, side of the pharyngotympanic tube was derived from the CNC as well (Fig 6C and 6D and S2 Fig). Moreover, the posterior attachment of the PLP was anchored in a mesoderm-derived pharyngeal wall, constituting the posterior border of the CNC-derived mesenchyme domain of the pharynx (Fig 6E and 6F). The pharyngeal constrictor muscles were partially embedded in neural crest-derived mesenchyme, and the pharyngeal wall constituted the posterior border of the neural crest contribution to the craniofacial mesenchymal tissues (Fig 6G and 6H). Thus, the pharyngeal wall may represent an interface between CNC-derived mesenchyme and mesoderm-derived mesenchyme.

## Discussion

In this study, we describe for the first time the anatomy and myogenesis of all four muscles of the soft palate in the mouse, including their relationship to the surrounding nerves, vasculature and neural crest cell-derived mesenchyme. We also highlight the similarities and differences between mouse and human palates. Most of our knowledge about palate formation comes from mouse genetic studies, especially through tissue-specific inactivation of genes of interest. Mice and humans share many similarities during their craniofacial development, making the mouse an excellent model for studying the molecular events orchestrating palatogenesis [2, 12]. However unlike humans, mice do not exhibit a uvula. Also, whereas humans are only able to breathe and suckle at the same time during the first year of life, rodents maintain this ability throughout their lifetime because their epiglottis is located above the soft palate. In addition, the mouse pharynx is relatively straight and narrow whereas the human pharynx is flexed and wide. Previously, it remained unclear whether these differences result from dissimilarities in the arrangement of the soft palate muscles, making the use of mouse models questionable to study posterior palate and pharynx formation. Although the rodent TVP and LVP have been previously described as similar to those of humans [15, 22], the PLG and PLP had yet to be properly characterized. Our study indicates that the orientation and attachment of each muscle of the palate are the same in mice and humans, thus validating the murine model for the study of palate myogenesis and associated defects. In both species, the soft palate fuses along the antero-posterior axis, followed by the disintegration of the medial edge epithelium.

During fusion, the soft palate muscles are partially differentiated. Our analysis during palatogenesis showed that the TVP differentiates first, as in humans [5, 23]. Previously it has been debated whether the TVP and TT are two separate muscles or form a complex [23]. Our data suggest that both muscles express differentiation markers and are innervated and vascularized at E13.5, but the density and general organization of the fibers of the TT suggest that it matures faster and independently of the TVP. Previous studies have also suggested that the TVP originates from a blastema of masticatory muscles and thus could act early in development to initiate movements at the level of the meckelian articular complex, before the formation of the temporomandibular joint [24]. These movements may promote the withdrawal of the tongue and the elongation of the mandible, allowing the palatal shelves to elevate. In this regard, we observed a close proximity and similar orientation between the TVP and medial pterygoid muscles in the vertical palatal shelves of E13.5 samples.

Our data suggests that during palatal fusion each palatal shelf contains a differentiated myocyte population in the lateral regions of the LVP. A few hours after fusion, the midline region of the LVP and PLP remains undifferentiated, but the differentiation process progresses from the lateral regions to the midline, forming a continuous functioning muscle by newborn stage. This finding could have important implications in clinical practice and tissue regeneration if cleft palate patients still possess this putative myoblast subpopulation in their unfused soft palatal shelves. It remains unclear whether the process of fusion promotes muscle formation, because cleft soft palate typically exhibits reduced muscle mass and atrophic LVP, associated with a sagittal reorientation of the muscle fibers, attached anteriorly to the posterior end of the hard palate [25, 26]. These defects make soft palate repair challenging, especially considering the low muscle volume, insufficient intrinsic regeneration capacity of cleft muscle and strong potential for fibrosis [22].

The nerves and vasculature are important regulators of organogenesis and tissue homeostasis in the craniofacial region [20, 21]. The TVP is innervated by the medial pterygoid nerve, a branch of the mandibular nerve (V3) arising from the trigeminal ganglion (CN V). The source for the innervation of the LVP is more controversial. It is classically considered to be innervated by the vagus nerve (CN X) [27], but has also been proposed to receive innervation from the glossopharyngeal nerve (CN IX) [28, 29]. The soft palatal shelves are innervated by the lesser palatine (V2) and glossopharyngeal nerves and are supplied with blood by the lesser palatine artery. We analyzed the pattern of this neuro-vascular component in relation to the muscles during palatogenesis. Strikingly, we found that most of the neuro-vasculature was located in the oral side of the palate, complementing the pattern of differentiated muscles. Whether this pattern reflects a role of innervation and vasculature in guiding myogenesis, migration and/or differentiation of myoblasts remains to be determined.

Neural crest cells represent a population of multipotent cells originating from the dorsal side of the neural tube and giving rise to a wide array of different tissues during embryonic development in vertebrates [30, 31]. Most of the facial structures are derived from the CNC [32]. CNC cells regulate the formation of tongue muscle, mandibular muscles and teeth via tissue-tissue interactions [33, 34]. In the posterior palate, the CNC cells populate the palatal mesenchyme prior to myogenic progenitor migration into the palate. Later on, the CNC-derived aponeurosis is formed at the level of the TVP [16]. Previous work from our group demonstrated that *Tgfbr2* expression is required in the palatal shelf epithelium to activate Wnt/β-catenin signaling in the CNC-derived mesenchyme and promote myogenesis [15]. Thus, soft palate myogenesis may also be regulated via tissue-tissue interactions during development. Through cell lineage tracing utilizing the *Wnt1-Cre* line, a marker specific for pre-migratory neural crest cells [35], we found that all soft palate muscles are embedded in CNC mesenchyme at E15.5. Moreover, there is a network of CNC-derived mesenchymal fibroblasts in the developing soft palate that persists through adulthood. Previous studies have suggested that the early specification of cranial muscle is CNC-independent, but later migration, patterning and differentiation of those precursors require the CNC [36], and this is likely to be the case for the soft palate as well.

Finally, our study shows that the pharyngeal wall may represent an interface between CNC- and mesoderm-derived mesenchyme. The nasal side of the pharyngotympanic tube and the pharyngeal constrictor muscles are mainly mesoderm-derived whereas the oral side and the PLP attachment to the pharyngeal wall are CNC-derived. Other stages of *Wnt1-Cre;R26R* ^*fl/+*^ expression patterns are reported in the Jackson Lab Cre Expression Data [37]. Consistent with our results, they show a clear boundary between CNC- and mesoderm-derived mesenchyme at E12.5. As development proceeds, the CNC domain expands posteriorly and integrates within the mesoderm-derived tissue. Different origins could explain the absence of fusion between the medial posterior palate and the pharyngeal wall, an opening later known as the oropharynx, as well as the maintenance of the pharyngotympanic tube. Thus, we propose this region may be a useful model to study the appearance and maintenance of tissue boundaries during development.

## Supporting Information

S1 Fig Innervation extends to the medial edge of the palatal shelves during soft palate fusion. **(A-D)** MHC (green) and β3-tubulin (red) co-immunostaining of transverse sections of E14.5 mouse soft palates from cranial (A) to caudal (D). Arrows indicate the nerve fibers at the tip of the fusing palatal shelves. LC: longus capitis, NP: nasopharynx, OP: oropharynx, PC: pharyngeal constrictor muscles, PLG: palatoglossus, PTT: pharyngotympanic tube, T: tongue, TVP: tensor veli palatini. The schematic drawing indicates the orientation and the position of each section. Scale bars: 200μm.
(TIF)

S2 Fig Dual origin of the mesenchyme surrounding the pharyngotympanic tube. MHC (red) immunostaining of soft palates from newborn *Wnt1-Cre;Zsgreen* ^*fl/fl*^ mice. Arrows indicate the mesenchyme surrounding the pharyngotympanic tube, which is CNC-derived on the oral side and mesoderm-derived on the nasal side. LVP: levator veli palatini, NP: nasopharynx, OP: oropharynx, PTT: pharyngotympanic tube. The schematic drawing indicates the orientation and the position of each section. Scale bar: 200μm.
(TIF)
