# Supplementary figures and images for: A Comprehensive Study of Soft Palate Development in Mice
Source: PLoS One. 2015 Dec 15;10(12):e0145018. doi: 10.1371/journal.pone.0145018 (PMC4687642; doi:10.1371/journal.pone.0145018)

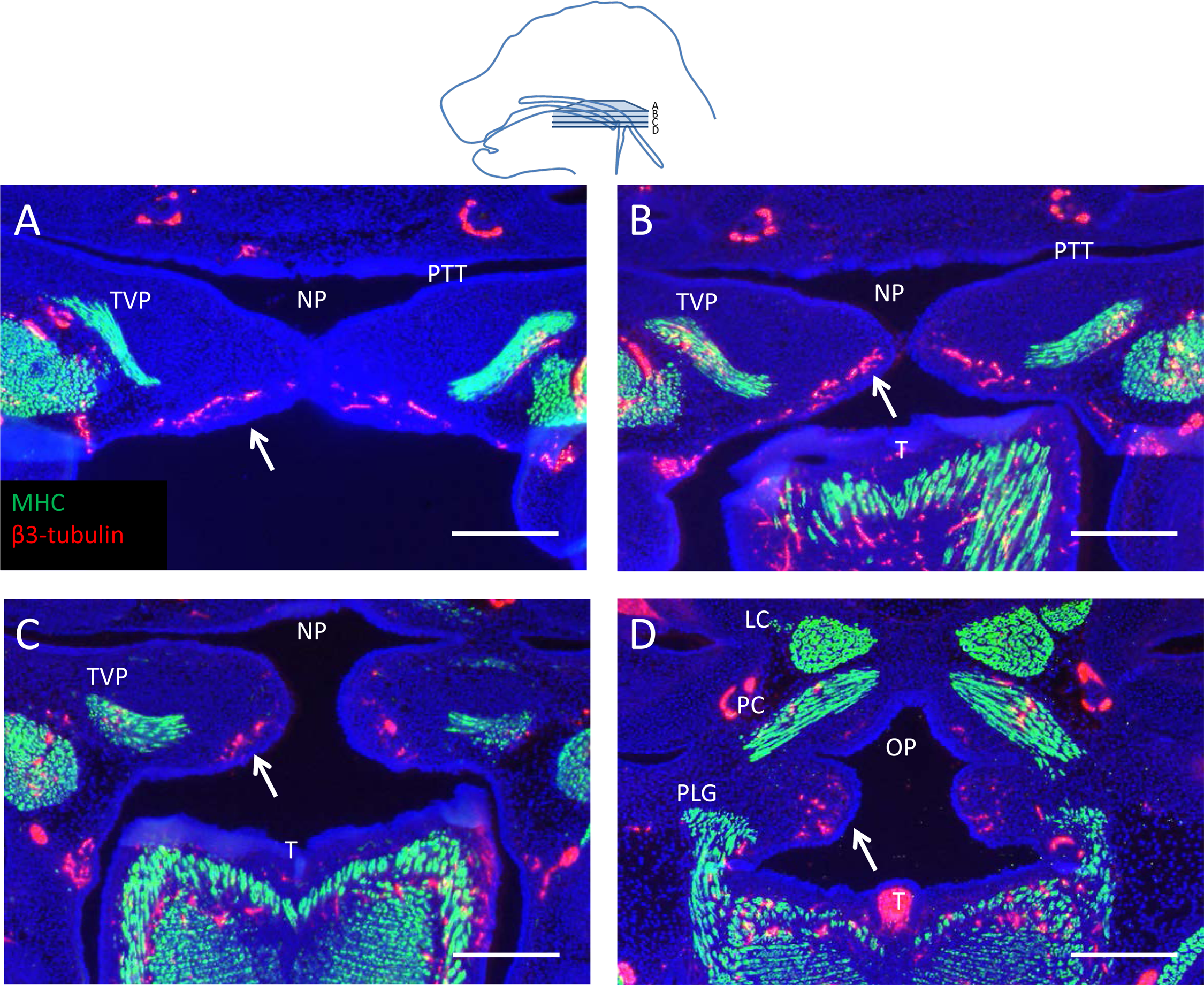

Supplement: S1 Fig — (A-D) MHC (green) and β3-tubulin (red) co-immunostaining of transverse sections of E14.5 mouse soft palates from cranial (A) to caudal (D). Arrows indicate the nerve fibers at the tip of the fusing palatal shelves. LC: longus capitis, NP: nasopharynx, OP: oropharynx, PC: pharyngeal constrictor muscles, PLG: palatoglossus, PTT: pharyngotympanic tube, T: tongue, TVP: tensor veli palatini. The schematic drawing indicates the orientation and the position of each section. Scale bars: 200μm. (TIF) [file pone.0145018.s001.tif]

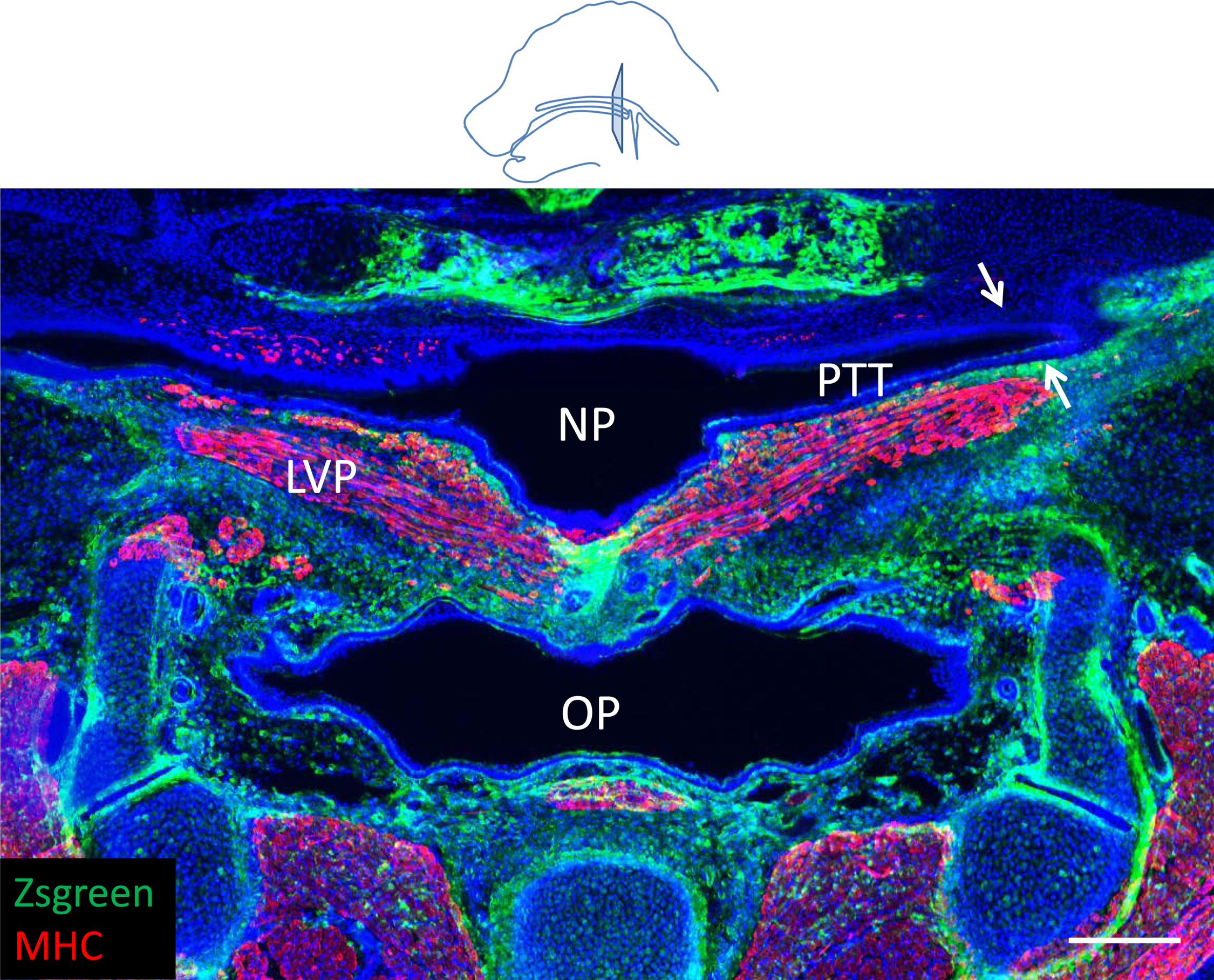

Supplement: S2 Fig — MHC (red) immunostaining of soft palates from newborn Wnt1-Cre;Zsgreen fl/fl mice. Arrows indicate the mesenchyme surrounding the pharyngotympanic tube, which is CNC-derived on the oral side and mesoderm-derived on the nasal side. LVP: levator veli palatini, NP: nasopharynx, OP: oropharynx, PTT: pharyngotympanic tube. The schematic drawing indicates the orientation and the position of each section. Scale bar: 200μm. (TIF) [file pone.0145018.s002.tif]
